# Cancer Survival Data Representation for Improved Parametric and Dynamic Lifetime Analysis

**DOI:** 10.3390/healthcare7040123

**Published:** 2019-10-28

**Authors:** Lode K.J. Vandamme, Peter A.A.F. Wouters, Gerrit D. Slooter, Ignace H.J.T. de Hingh

**Affiliations:** 1 Department of Electrical Engineering, Eindhoven University of Technology, 5612AE Eindhoven, The Netherlands; 2 Department of Surgical Oncology, Máxima Medical Center, 5504DB Veldhoven, The Netherlands; 3 Department of Surgery, Catharina Hospital, 5623EJ Eindhoven, The Netherlands

**Keywords:** parametric survival model, hazard rate, dynamic lifetime, cancer survival data, statistical distributions

## Abstract

Survival functions are often characterized by a median survival time or a 5-year survival. Whether or not such representation is sufficient depends on tumour development. Different tumour stages have different mean survival times after therapy. The validity of an exponential decay and the origins of deviations are substantiated. The paper shows, that representation of survival data as logarithmic functions visualizes differences better, which allows for differentiating short- and long-term dynamic lifetime. It is more instructive to represent the changing lifetime expectancy for an individual who has survived a certain time, which can be significantly different from the initial expectation just after treatment. Survival data from 15 publications on cancer are compared and re-analysed based on the well-established: (i) exponential decay (ii) piecewise constant hazard (iii) Weibull model and our proposed parametric survival models, (iv) the two-τ and (v) the sliding-τ model. The new models describe either accelerated aging or filtering out of defects with numerical parameters with a physical meaning and add information to the usually provided log-rank *P*-value or median survival. The statistical inhomogeneity in a group by mixing up different tumour stages, metastases and treatments is the main origin for deviations from the exponential decay.

## 1. Motivation and Objective

Kaplan-Meier curves provide a parameter-free statistical estimator for the survival function and are therefore attractive for application in cancer treatment studies [1]. From the curves, the median survival time, *T*_1/2_, can be retrieved. In medical literature, the effectiveness of treatments is expressed in terms of this value. The main drawback is the lack of discrimination in case life expectancy changes upon the time passed since treatment. To demonstrate the effectiveness of novel targeted therapies, there is a need to extract more detailed information from survival data than by providing *T*_1/2_ only. This paper provides means to evaluate the dynamics in the life expectancy in terms of simple but effective mathematical models. The added value of such approach is its capability to account for the consequences of an underlying mechanism, which may cause deviations from a pure exponential behaviour. This is relevant information, both for judging and comparing treatments as well as for communication of useful information to patients.

Graphing out Kaplan-Meier (KM) curves as the logarithm of the survival function *S*(*t*) versus a linear time scale *t* (log-lin) improves the lifetime data presentation, by providing figures of merit for a cancer treatment and prognosis, which can directly be observed from the graphs. From the log-lin KM curve, it can be observed whether the survival function decays with a single time constant. If not, the initial dynamic lifetime *τ_dyn_* (0) differs from the value at a later observation time *τ_dyn_* (*t_max_*). Deviations from an exponential decay show up as deviations from a straight line. This can be interpreted in terms of a dynamic lifetime *τ_dyn_* which evolves with time. Such dynamics change the lifetime expectancy of an individual patient and providing an over-all medium value can be misleading. A distinction between a short- and long-term dynamic lifetime, *τ_dyn_* (0) and *τ_dyn_* (*t_max_*), is more informative. As mentioned in [1]: The *τ_dyn_* (*t*) is about the instantaneous potential lifetime for a person “not to fail” after a survival time *t*. The hazard function *h* (*t*) gives the instantaneous potential for a person per unit time for an event (dead or relapse) to occur, after being alive for a time *t*.

Decay processes with different physical origins show exponential decay, *S* (*t*) = e^−t/^^τ^ with *τ* the characteristic time. This implies a constant hazard rate (or risk), *h* (*t*) = 1/*τ*, independent of survival time. Only then the median time relates to the characteristic time, which is the average lifetime, as *τ* = 1.44 × *T*_1/2_. In physics and engineering, it is common practice to present a nonlinear dependency in appropriate scales to obtain a straight line in order to check an underlying hypothesis. We have an advantage by following this tradition of “appropriate” scales. To the best of our knowledge, log-lin scales are not used for KM curves in oncology and are not advocated in recent or old textbooks on the topic, see e.g. [1,2].

The objective is to illustrate the advantages of graphing out *S* (*t*) in log-lin and the analysis in short- and long-term lifetime *τ_dyn_* (0) and *τ_dyn_* (*t_max_*) with curve fitting to a parametric survival model. If *τ_dyn_* (*t*) is about constant, then the group of patients is statistically homogeneous with the same (low) tumour stage and a homogeneous treatment. If *τ_dyn_* (0) and *τ_dyn_* (*t_max_*) are quite different, the modelling should account for the dynamics in the lifetime expectancy. The first contribution of this paper is the exploration of the significance of including a dynamic lifetime expectancy, based on 15 published studies (Section 3.1). Several options for a time-varying lifetime expectation are implemented and compared. To this end, well-established methods for survival analysis are employed: exponential, Weibull and piecewise linear distributions. The second contribution of the paper is the proposal of two new models: two-τ (Section 2.2.1) and sliding-τ (Section 2.2.2). These models involve numerical parameters with a physical meaning. Their values add information to the usually provided log-rank *p*-value. This is exemplified for a few selected studies, where survival expectancies after e.g., 1, 3, and 5 years are compared. The capability to formulate hypotheses based on the proposed models is a third contribution of this paper (Section 3.2).

## 2. Dynamic Lifetime Analysis 

Deviations from constant risk are directly visualized in log-lin plots of the survival function with time. For quantifying these deviations, models can be employed where the dynamic lifetime is parameterized. This section introduces techniques employed in this paper for the re-analysis of 15 published cancer survival studies to analyse potential dynamics in the lifetime expectancy. Section 2.1 briefly summarizes a few aspects related to such dynamics for the survival function. In Section 2.2, options are presented which account for evolving lifetime expectancy.

### 2.1. The Survival Function

The number of survivors *N* (*t*) decreases with time *t* from the initial number *N* (0). The reduction in survivors per interval d*t* is the rate d*N* (*t*)/d*t*. The random variable is survival time until an event occurs, e.g. death or relapse. Statistical phenomena in time are described by rate equations:

(1) $$\frac{dN\left(t\right)}{dt}=\frac{-1}{\tau \left(t\right)}N\left(t\right)$$

The characteristic time *τ* for the decay process can be constant or may change over time. The former case is referred to as the Poisson process and states that the probability of a casualty per person and per unit time does not change. In this case, the solution is an exponential decay:

(2) $$N\left(t\right)=N\left(0\right)e^{-\frac{t}{\tau }}\;\mathrm{and}\;S\left(t\right)\equiv \frac{N\left(t\right)}{N\left(0\right)}=e^{-\frac{t}{\tau }}$$

and at time *T*_1/2_ = *τ*ln2, the surviving fraction is halved. The survival after a treatment is a statistical process. The survival function *S* (*t*) provides the surviving fraction as a function of time. In medical treatment, *τ* may either increase or decrease with time. This leads to the definition of a dynamic characteristic time *τ_dyn_* which changes depending on the time elapsed after treatment, in terms of the hazard function *h* (*t*):

(3) $$h\left(t\right)\equiv \frac{1}{\tau_{dyn}\left(t\right)}=-\frac{d\ln S\left(t\right)}{dt},\;\mathrm{giving}\;h\left(t\right)=\frac{-1}{S\left(t\right)}\frac{dS\left(t\right)}{dt}$$

Multiple uncorrelated hazards can be added. As an example, consider a hazard function *h_c_* = 1/*τ_c_* from cancer treatment for which the data is impeded by another, independent, hazard cause *h_o_* = 1/*τ_o_*. The total hazard *h_co_* = 1/*τ_co_* is given by their sum, and the cancer-specific dynamic time constant is:

(4) $$h_{co}\left(t\right)=h_{c}\left(t\right)+h_{o}\left(t\right)\;\mathrm{resulting}\;\mathrm{in}\;\;\tau_{c}=\frac{\tau_{co}}{1-\tau_{co}/\tau_{o}}$$

To prevent misjudgment of the risk, one needs to correct the observed time constant *τ_co_*. Data for *τ_o_* can be obtained in life tables at different ages. Then, one can employ the relative survival, being the ratio between the survival amongst cancer patients and survival of a representative control population. It eliminates the influence by other hazards:

(5) $$S_{c}\left(t\right)\equiv \frac{S_{co}\left(t\right)}{S_{o}\left(t\right)}\;\mathrm{implying}\;S_{c}\left(t\right)=\frac{e^{-t/\tau_{co}}}{e^{-t/\tau_{o}}}=e^{-t/\tau_{c}}$$

**Figure 1:**
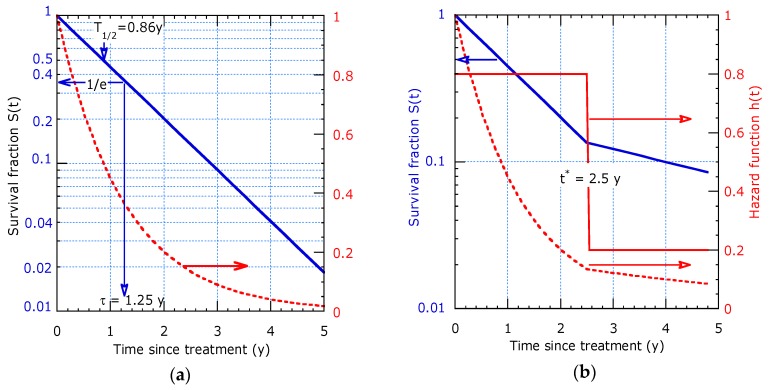
Exponential decay presented as: (**a**) Logarithmic plot providing a straight solid line for constant hazard (left scale) and as linear plot (dashed line, right hand scale); (**b**) Piecewise linear decay solid line on logarithmic scale (left) and dashed line on linear scale (right) with sudden transition at time t* = 2.5 y from a discontinuity in the hazard (right, *h* = 0.8 y^−1^ for *t* < t* and *h* = 0.2 y^−1^ for *t* > t*).

Only for the Poisson process, the survival function is a pure exponential function. Such behaviour can conveniently be represented as log-lin plot. As shown in Figure 1a, the relationship is a straight line with a slope proportional to -1/*τ*, which cannot be observed in the linear plot. Only for a constant decay rate the dynamic lifetime is equal to the characteristic time and has a fixed ratio with respect to the median survival time: *τ*/*T*_½_ = 1.44. The exponential decay behaves initially linear, since *S* (*t*) = e^−*t/τ*^ ≈ 1−*t*/*τ* for times appreciably shorter than the characteristic time. An extension is the piecewise constant hazard model, as advocated e.g., in [2], where the characteristic time constant suddenly changes beyond a transition time t*. This results in two distinct slopes around t* in a logarithmic representation as shown in Figure 1b. Deviations from the pure Poisson process will be discussed in the following subsections with focus on proper graphical visualization and computer curve fitting to the parametric survival model.

### 2.2. Modeling of Dynamic Lifetime

The piecewise constant hazard model can be seen as a time-dependent hazard model with only two distinct values. The discontinuity in the hazard function is an inherent disadvantage. A flexible decay function is implemented as a shape parameter *k* in the Weibull distribution:

(6) $$S\left(t\right)=e^{-{\left(t/\tau \right)}^{k}}$$

With (3) the shape factor *k* can lead to an increasing (*k* < 1) or decreasing (*k* > 1) value for dynamic lifetime:

(7) $$\tau_{dyn}\left(t\right)=\frac{\tau }{k}{\left(\frac{t}{\tau }\right)}^{1-k}$$

Weibull distributions are widely used in reliability studies, but for lifetime modelling they have the nasty property that unless *k* = 1 (Poisson distribution), the initial dynamic time constant is either zero (*k* < 1) or infinity (*k* > 1). The shape factor *k* has no physical meaning in the sense of a simple relationship with short or long term dynamic lifetime, and *τ* is only indirectly related to the mean lifetime. The proposed alternatives are the two-τ model (Section 2.2.1) and the sliding-τ model (Section 2.2.2).

#### 2.2.1. Model with Two Distinct Decay Times

As an illustration, consider an inhomogeneous group with a majority population (A: fraction *p*) having a decay time *τ*_1_ and a minority population (B: fraction 1-*p*) with higher decay *τ*_2_. Group B could have a lower tumour stage or a better immune and repair system to tackle the disease compared to group A. The total survival function is a weighted sum of both populations:

(8) $$S\left(t\right)\equiv A\left(t\right)+B\left(t\right)=pe^{-t/\tau_{1}}+\left(1-p\right)e^{-t/\tau_{2}}$$

The dynamic lifetime *τ_dyn_* depends on fraction *p* according to:

(9) $$\tau_{dyn}\left(t\right)=\frac{pe^{-t/\tau_{1}}+\left(1-p\right)e^{-t/\tau_{2}}}{\frac{p}{\tau_{1}}e^{-t/\tau_{1}}+\frac{\left(1-p\right)}{\tau_{2}}e^{-t/\tau_{2}}}$$

**Figure 2:**
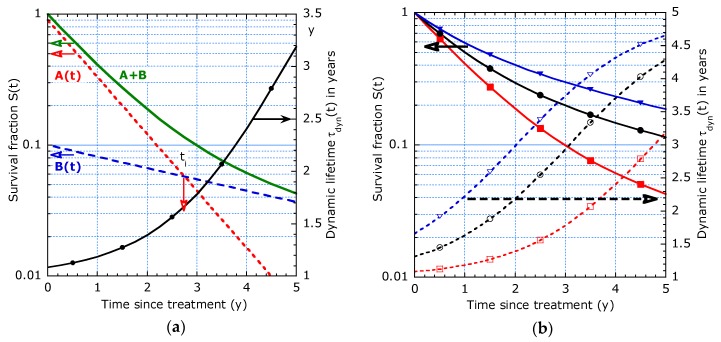
Example of the survival function (left scale) and dynamic lifetime (right scale) for the two-τ model with *τ*_1_ = 1 y and *τ*_2_ = 5 y: (**a**) Combined and separate subpopulations A (*p* = 0.9) and B with intersecting survival at *t_i_*; (**b**) Effect of different majority fractions with *p* = 0.5 (triangles), *p* = 0.7 (circles) and *p* = 0.9 (squares).

As shown in Figure 2a, though the components *A*(*t*) and *B*(*t*) are straight lines, their summation *S*(*t*) is not. The dynamic lifetime increases from an initial value depending on *p*:

(10) $$\tau_{dyn}\left(0\right)=\frac{1}{\frac{p}{\tau_{1}}+\frac{\left(1-p\right)}{\tau_{2}}}$$

Its slope continuously changes around *t_i_* where the individual *A*- and *B*-functions intersect. This is an essential distinction from the piecewise linear model. The given example with *τ*_1_ = 1 y, *τ*_2_ = 5 y and *p* = 0.9 also points out the increasing lifetime expectation with survival time. Figure 2b shows the effect of a majority fraction *p* both in terms of the survival function and dynamic time. Detecting an inhomogeneity in the population from inspection of such graphs requires that the involved characteristic times differ significantly (e.g., factor 4), the minority population is clearly smaller (e.g., 1-*p* < ¼) and the follow-up time of the group clearly exceeds *τ*_1_ (e.g., > factor 3). Mixing up patients with different tumour stages increases the statistical inhomogeneity of the group and is the main origin for deviations from exponential decay. The model can be refined by increasing the number of subgroups, but interpretation becomes hard. Alternatively, one can adopt the “sliding-τ” approach presented in the next subsection.

#### 2.2.2. Model with Continuous Evolving Decay Time

Due to a mix of high and low tumour stages in a group, “filtering out of defects” can occur with a decrease in *h* (*t*) with time. Pooling tumour stages makes the cohort statistically inhomogeneous. The origin of filtering out can be explained by the early deceased members of the group with a higher risk (higher tumour stage). As time goes by, the percentage of patients with a lower tumour stage increases. Therefore, studies selecting on the type of metastasis, but with a mixture of high and low tumour stages, can result in filtering out. On the opposite side, accelerated aging imposes an increase in failure rate at later times. Accelerated aging may be induced by a very aggressive tumour, chemo or radiation therapy, age or a remainder of tumour cells above a critical threshold. This results in a fast relapse. The sliding characteristic time can be formulated as *τ* (t) = *τ*_0_ (1 + *bt*) resulting in:

(11) $$S\left(t\right)=e^{-t/\tau_{0}\left(1+bt\right)}\;\mathrm{and}\;\tau_{dyn}\left(t\right)=\tau_{0}{\left(1+bt\right)}^{2}$$

The parameter *τ*_0_ is the initial lifetime *τ_dyn_* (0), which either increases (*b* > 0) or decreases (*b* < 0; note that only values with *bt* > −1 are meaningful).

The sign of a second derivative of ln *S*(*t*) with respect to time is given by the sign of *b*:

(12) $$\frac{d^{2}\ln S\left(t\right)}{dt^{2}}=\frac{2b}{\tau_{0}{\left(1+bt\right)}^{3}}\;\mathrm{with}\;\left(1+bt\right)>0\;\;\;\begin{array}{c} b<0:\;\mathrm{concave}\;\mathrm{curve}\\ b>0:\;\mathrm{convex}\;\mathrm{curve}\; \end{array}$$

**Figure 3:**
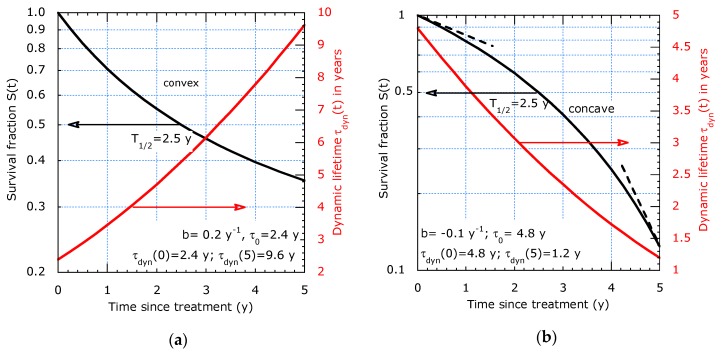
Survival (left scale) and dynamic lifetime (right scale) example of the sliding-τ model: (**a**) Filtering out of defects (convex curve): *b* = 0.2 y^−1^ and *τ_dyn_* (0) = 2.4 y, *τ_dyn_* (5) = 9.6 y; (**b**) Accelerated aging (concave curve): *b* = −0.1 y^−1^, *τ_dyn_* (0) = 4.8 y and *τ_dyn_* (5) = 1.2 y.

It decides on a convex or concave descending survival curve in a logarithmic representation, which can immediately be observed from Figure 3. The two-τ model results also in convex curves, see Figure 2. The convex curve in Figure 3a corresponds with an increasing dynamic lifetime, whereas the concave curve in Figure 3b points to a reducing dynamic lifetime. In the examples in Figure 3a,b, the median T_½_ is 2.5 y but the curves have quite different *τ_dyn_* (*t_max_*) and *τ_dyn_* (0): *T*_1/2_ is not a proper predictor for both initial (*t* = 0) and long-term (*t* = 5 y) lifetime expectancies. The tangent lines, indicated by dashed lines in Figure 3b at *t* = 5 y and 0 y, illustrate the difference in short and long term lifetime expectancy. The fitted curve with (11) interpolates in between. From points (*t_i_*; *S_i_*) on the tangent line, we can calculate the slopes and the initial and long term *τ_dyn_* as follows:

(13) $$\tau_{dyn}\left(0\right)=\frac{t_{2}}{\ln\left(1/S_{2}\right)}\;\;\mathrm{and}\;\;\tau_{dyn}\left(t_{max}\right)=\frac{t_{2}-t_{1}}{\ln\left(S_{1}/S_{2}\right)}.$$

The ratio, *R* = *τ_dyn_*(*t_max_*)/*τ_dyn_*(0), depends on *bt_max_*

(14) $$R=\frac{\tau_{dyn}\left(t_{max}\right)}{\tau_{dyn}\left(0\right)}={\left(1+bt_{max}\right)}^{2}.$$

## 3. Re-Analysis of Cancer Survival Data from Literature

Publications on cancer with survival data are reinterpreted in terms of potentially additional information on evolving lifetime expectation from the data [3,4,5,6,7,8,9,10,11,12,13,14,15,16,17]. The different methodologies presented in Section 2 are applied and their results are presented as log-lin graphs in Section 3.1. Section 3.2 elaborates on the merits of employing dynamic lifetime analysis, in addition to providing only median values or survival fractions after a period of time. Either numerical data in tables served as input or data extracted from linear KM plots. The plots are digitized manually by sampling at equidistant time instances. Per plot, about 10 points are taken, if possible. Six points per curve are considered as a minimum required for a reliable curve fit with two parameters. The curve fit analysis is performed by means of KaleidaGraph-Synergy Software [18]. The shape of curves in the logarithmic representation is indicative for the choice of the appropriate model and curve fit.

Deviations from exponential decay result in differences in long and short-term dynamic lifetimes. To judge such a difference, the ratio *R* = *τ_dyn_* (*t_max_*)/*τ_dyn_* (0) is calculated from (14). For the re-analysed data, satisfying either the Poisson exponential decay or the sliding-τ model, the corresponding figures show the curve fits together with the model parameters: *τ* for the Poisson; *τ*_0_, *b* for the sliding-τ model; *τ* and *k* for the Weibull model; and *τ*_1_, *τ*_2_ and *p* for the two-τ model. If the Poisson model is applicable (*R* is one), the median value *T*_1/2_ is supplied. In a few cases, when the Poisson model does not apply (*R* significantly differs from one), tangent lines (dashed) are provided to indicate initial, *τ_dyn_* (0), or late, *τ_dyn_* (*t_max_*), dynamic lifetimes based on the slopes in the data besides the fitted curves. In a few studies, the two-τ, Weibull model and piecewise linear model are compared with the sliding-τ model.

### 3.1. Case Studies from Literature

Data from 15 publications are re-analysed in dynamic lifetimes. The model parameter values in the insets of the figures are provided with the standard deviation (between brackets) indicating their confidence bounds. Lifetimes are expressed in years or months, abbreviated as ‘y’ and ‘m’.

1. Study by van Gestel et al. [3] on colorectal cancer (CRC), mainly with tumour stage III.

**Figure 4:**
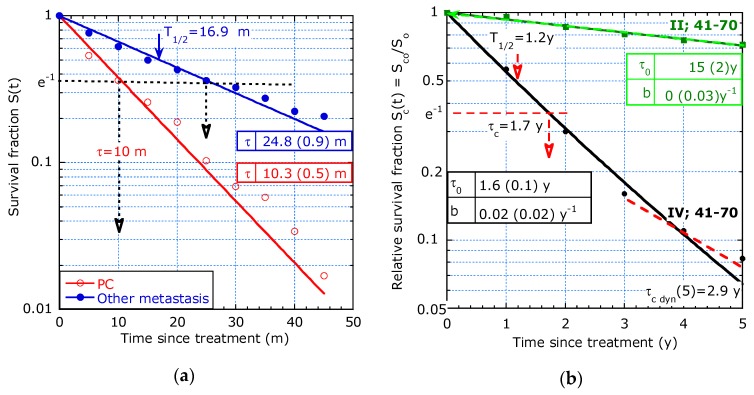
Survival estimation with exponential decay or a sliding-τ model with *b* ≈ 0: (**a**) After curative treatment of CRC (mainly stage III) with PC (circles) and other metastases (dots) ([3], Figure 2); (**b**) Relative survival of patients in the age group 41–70 years old, stage II (squares) and IV (dots) ([4], Figure 1).

Figure 4a compares the survival since diagnosis of peritoneal carcinomatosis (PC) with other metastases among colorectal cancer patients diagnosed between 2003 and 2008 after curative treatment: PC (circles); other metastasis (dots). The KM data show straight lines on a logarithmic scale implying a curve fit with an exponential decay. The fit results in *τ* = 25 m for other metastasis and *τ* = 10 m for PC. The *τ*-values can be verified by the intersection of the straight line with *S*(*t*) = 1/e. This is more informative than a p-value of 0.001 in a log-rank test in [3].

2. Study by Orsini et al. [4] on 37,056 rectal cancer patients in the Netherlands between 1989 and 2010 according to the stage and stratified by age (until 40 years and 41–70 years).

Figure 4b shows the (re-analysed) relative survival rate (RSR) of an age group of 41–70 years old, only stage II and IV patients are discussed. The sliding-τ model is applied. However, the parameter *b* is zero within its confidence bound indicating that the Poisson model holds for stage II. Stage IV shows an initial lifetime expectancy *τ*_0_ = 1.6 y, increasing to 2.9 y after 5 years by analysing the slope of the tangent line.

The benefit from an early diagnosis is clear: *τ*_0_ = 15 y for stage II and *τ*_0_ = 1.6 y for stage IV. The higher the tumour stage, the larger its size and its surface and the higher the tumour cell leakage and that may explain the lower average lifetime and the benefit of an early diagnosis.

3. Study by Stelzner et al. [5] on CRC selected by stage, with the aim to justify a preference for relative survival as a standard measure for surgical audit and quality control.

**Figure 5:**
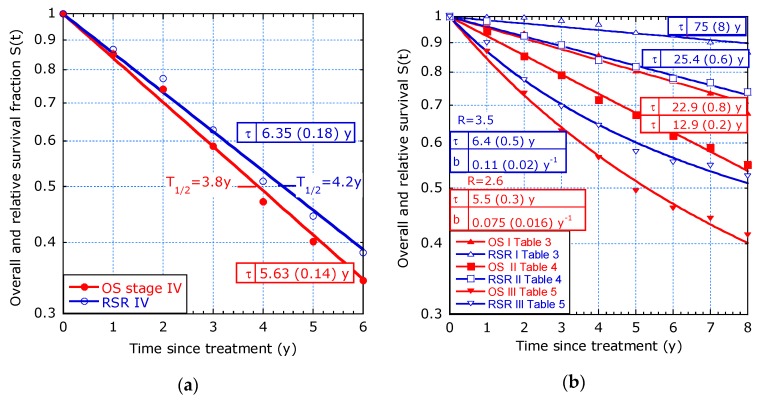
Rectal cancer selected with respect to tumour stage: (**a**) Exponential decay is applied to OS (red) and RSR (blue) for stage IV ([5], Table 6); (**b**) OS and RSR ([5], Tables 3 and 4). Stages I, II fit the exponential decay. The time-dependent lifetime in stage III fits the sliding-τ model.

Figure 5a shows the Poisson fit for stage IV. As expected, the overall survival (OS) has a slightly lower characteristic time than the relative survival rate (RSR). Figure 5b shows that stages I and II can be described with a single exponential, but stage III needs a time-dependent hazard. For stage III, the ratios between long- and short-term expectations are *R* = 3.5 for RSR and 2.6 for OS. The stage III group seems to be statistically less homogeneous than the groups with stage I, II and IV. The “filtering out defects” model is often applicable for stage III and sometimes for stage IV, provided the observation time and the number of patients are large enough.

4. Study by Bollschweiler [6] on gastric cancer with patients having different tumour stages after gastrectomy with the aim to discuss the interpretation of KM survival plots.

**Figure 6:**
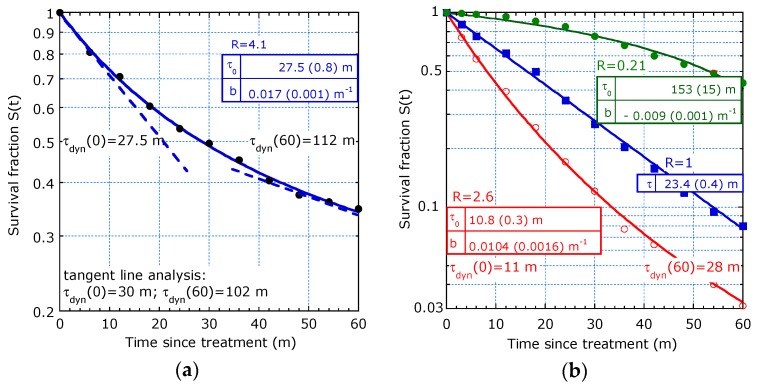
Decreasing and increasing hazards: (**a**) Mixture of tumour stages from gastric cancer modelled with sliding-τ in favour of filtering out defects with tangent lines at *τ_dyn_* (0) and *τ_dyn_* (*t_max_*) (dashed lines) ([6], Figure 1); (**b**) Sliding-τ model with accelerated aging (*b* < 0, dots), exponential decay (*b* ≈ 0, squares) and filtering out defects (*b* > 0, circles) ([7], Figure 3).

The survival plot in Figure 6a shows a clear deviation from a straight line. The values beyond 60 m in the original data are omitted because of a too low number of patients (only 9). The curve fits the sliding-τ model in support of “filtering out defects”, which is typical for a statistical inhomogeneous group. The dynamic lifetime increases from *τ_dyn_*(0) = 28 m to *τ_dyn_*(60) = 112 m as calculated from the fit parameters and it agrees with the analysis from the slopes of tangent lines. The *τ_dyn_*(0), *τ_dyn_*(60) and *R* = 4.1 are more informative than the characterization by a median survival time of 30 m (95% confidence interval 16.5–37.7 m) as mentioned in [6].

5. Study by ‘t Lam-Boer et al. [7] on OS depending on different surgical therapies of 1617 patients between 2004 and 2012 with CRC of stage IV and metastases confined to the liver only. The aim of their study was to investigate changes over time in the utilization of liver resections, as well as possible institutional variations.

The group with the resection of the primary tumour and liver metastases in Figure 6b (dots) shows a concave curve and fits the sliding-τ model with *b* < 0 (accelerated aging). The values *τ*_0_ = 153 m (15 m) and *R* = 0.23 are more informative than only *T*_1/2_ = 55 m as provided in [7]. The group with resection of primary tumour only (squares) fit the exponential decay with *τ* = 23.4 m (0.4 m). The group with no resection at all (circles) fits the filtering out defects.

The lack of statistical homogeneity in two groups is probably due to the fact that patients with metastases were diagnosed in different hospitals. The institutional variations were important. A factor two difference in chance of undergoing liver surgery between institutions is mentioned in [7]. This may explain the deviation from exponential decay.

6. Study from Razenberg et al. [8] on 4430 patients with CRC and PC treated with cytoreductive surgery (CRS) and hyperthermic intraperitoneal chemotherapy (HIPEC).

**Figure 7:**
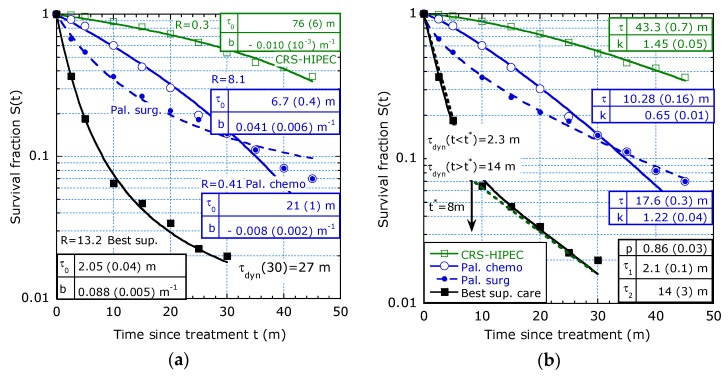
Comparison between models on OS of patients with PC of CRC origin: (**a**) Sliding-τ model applied to (i) CRS+HIPEC (open squares), (ii) palliative chemotherapy (circles), (iii) palliative surgery (dots), and (iv) best supportive care (full squares) ([8], Figure 2); (**b**) Weibull model applied on case: (i), (ii), and (iii). Case (iv) is fitted with the two-τ model and the piecewise linear model ([8], Figure 2).

Figure 7a shows four fits with the sliding-τ model and two with accelerated aging, (*b* < 0) (i) CRS+HIPEC (open squares) and (ii) palliative chemotherapy (circles). The latter group has 25% of patients of stage III and 46% of stage IV [8], which may explain the statistical inhomogeneity. Two groups fit the filtering out defects (*b* > 0): (iii) palliative surgery (dots) and iv) best supportive care (full squares). The palliative surgery has a lower median time than palliative chemo but the curve fit suggests that it has a higher long-term expectation. The best supportive care group consists of elected patients on objective criteria by the medical staff and a minority group of patients refusing further treatment. This may explain the inhomogeneity in the group with stage IV and the good fit with both models: the sliding-τ model in Figure 7a and also the two-τ model in Figure 7b. For comparison, Figure 7b also shows three fits to the Weibull model. An interpretation with the piecewise linear model (dashed lines) with t* = 8 m is also a possibility for best supportive care.

7. Study by Kuijpers et al. [9] evaluating results of CRS+HIPEC for PC of CRC origin in the Netherlands following a national protocol.

**Figure 8:**
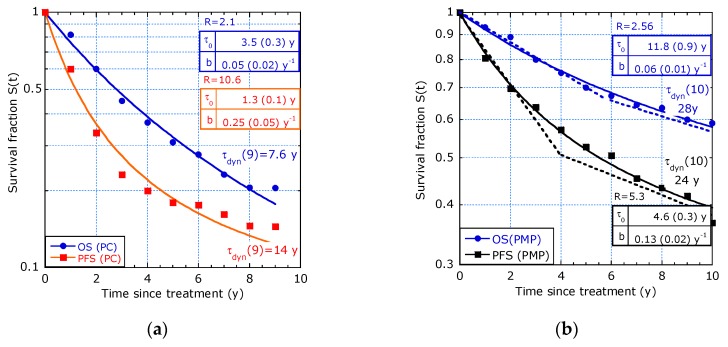
OS and PFS fitted to the sliding-τ model from ([9], Figure 2a,b): (**a**) Patients selected on PC; (**b**) Patients selected on PMP.

The patients were selected on PC and on pseudomyxoma peritonei (PMP) but in each group are patients with different tumour stages. In the 10-year survival estimation, the difference was investigated for 660 patients with PC and 300 patients with PMP in terms of OS (i.e., only death as event) and PFS (progression-free survival, i.e., tumour progression and death as events). The survival function in Figure 8a on PC and Figure 8b on PMP patients fits the sliding-τ model, providing increasing lifetime expectancy. The piecewise constant hazard model with a proper transition time is also possible (dashed lines in Figure 8b).

8. Study by Glehen et al. [10] on PC of digestive or primary origin with mixtures of tumour stages and treatments such as HIPEC and early postoperative intraperitoneal chemotherapy (EPIC).

**Figure 9:**
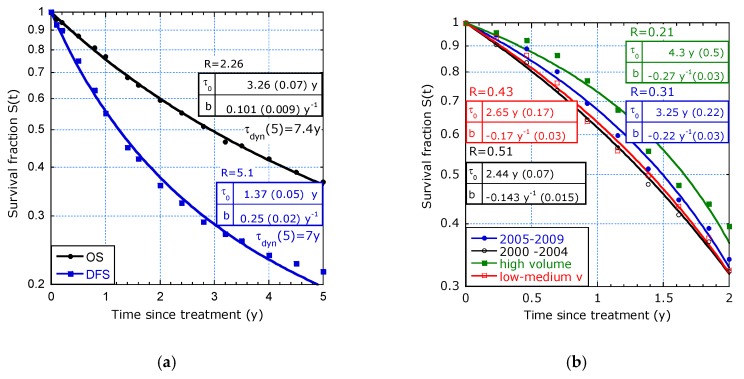
Filtering out of defects, *b* > 0 and accelerated aging, *b* < 0: (**a**) PC of digestive or primary origin with different tumour stages show for defect-free survival (DFS, squares) a ratio *R* ≈ 5.1 and for overall survival (OS) a ratio *R* ≈ 2.3 ([10], Figure 2); (**b**) Pancreatic cancer survival by period of diagnosis, 2005–2009 and 2000–2004 and by hospital volume show accelerated aging ([11], Figure 1a,b).

Figure 9a concerns a multi-institutional study in French-speaking countries of 1290 patients on curative treatment by CRS + EPIC or CRS + HIPEC. It shows a similar trend as observed in Figure 8. The increasing lifetime expectancy is typical for groups with mixtures of tumour stages and treatments (HIPEC or EPIC). As expected, its initial value is higher for OS than for defect-free survival (DFS). Remarkably, the long-term lifetime expectancies in OS for PC of CRC origin in [9,10] are similar and Figure 9a shows that *τ_dyn_* (*t_max_*) is about 7 y in both cases, OS and DFS.

9. Study from Gooiker et al. [11] on pancreatic cancer with the aim to analyse the impact of nationwide centralization of pancreatic surgery on long-term survival.

Figure 9b depicts the survival retrieved from the study [11] on 1639 patients on a 2-year survival estimation. The concave curves indicate accelerated aging, *b* < 0. The insets show different values for *τ*_0_, *b* and *R*. The systematic trend between the parameters is: the higher *τ*_0_, the smaller *b* (*b* < 0) and the lower *R* (*R* < 1). Surprisingly, in all cases holds a long-term lifetime expectancy, *τ_dyn_*(2) ≈ 1.1 y. No significant difference in survival was mentioned between the periods of diagnosis in the intervals 2005–2009 and 2000–2004 (*p* = 0.135). The difference between high and medium-low hospital volume is significant (*p* = 0.017). Parametric survival modelling quantifies the life expectancy with uncertainty margin, which may be more relevant in communication to patients.

10. Survival analysis from studies with KM plots hampered by limited data.

**Figure 10:**
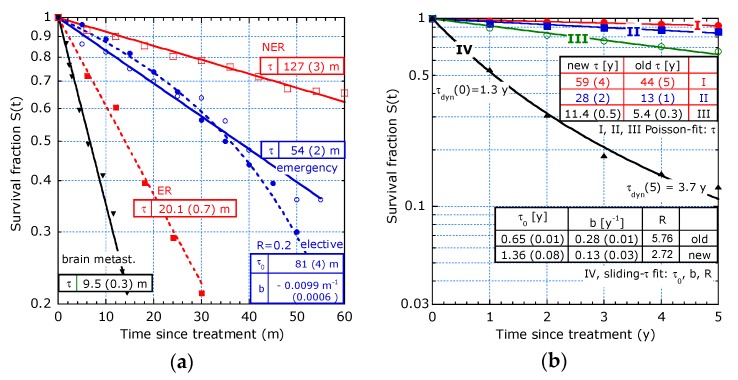
Smoothening of KM curves for studies on small populations and comparison between time period: (**a**) CRS + HIPEC study on elective (blue dots) and emergency (blue circles) patients from Figure 2 in [12], early recurrence (ER, red full squares) and non-early recurrence (NER, red open squares) from Figure 1 in [13], survival from brain metastases from Figure 2 in [14] (black triangles); (**b**) Time period study on CRC with stages I, II and III fit the Poisson model; stage IV fits the sliding-τ model, only period 2010–2014 (new) is shown, based on Figure 2a–d in [15] and period 1989–1994 (old) is compared with ‘new’ in the two insets.

The survival analysis at a low sampling rate and few patients results in a ‘staircase’ KM graph with broad and often deep steps. For Figure 10a, an interpolation procedure is used to smooth discontinuities. At a large step in the survival data at a specific time, the average before and after is assigned to that time. For a wide step between two consecutive times in the data, the survival value is attributed at the midway point between these times. This smoothing procedure is applied for three studies in [12,13,14] for Figure 10a.

Van Oudheusden et al. [12] investigated survival after CRS+HIPEC on only 149 patients between an elective and emergency group. The curve with the elective patients for CRS+HIPEC (dots) relates to accelerated aging. The emergency patients (circles) exhibit an exponential decay. The emergency group received HIPEC a few months after the emergency CRS. Perhaps this group is statistically more homogeneous than the elective group.

Kwong et al. [13] studied hepatocellular carcinoma on 167 patients. The early recurrence (ER) group (full squares) with only 40 patients and the non-early recurrence (NER) group (open squares) with 127 patients after hepatectomy fit the exponential decay. The characteristic times are 20 m and 127 m, respectively.

The study by Rades et al. in [14] investigated survival of patients with brain metastases from small-cell lung cancer on 157 patients after being irradiated for brain metastases. The sharp selection in the intermediate group results in exponential decay (triangles) with *τ* = 9.5 m.

11. Study by Brouwer et al. [15] on 25 years of incidence, treatment and outcome of CRC in five periods of diagnosis and only five data points per curve is shown in Figure 10b.

Data from a group of patients over the period 2010–2014 (new) are presented and compared with the period 1989–1994 (old). Stages I, II and III satisfy a Poisson behaviour. The data for stage I, II, and III show a clear improvement. The analysis in dynamic lifetime is more sensitive than the analysis in a 5-year survival percentage, *S* (5) as in [15]. For example, stage I: old *S* (5) = 91%, new 92% and in average lifetime: old 44 y and new up to 59 y. For stage III holds: old *S* (5) = 45%, new 68% and in *τ*-values: old 5.4 y, new 11.4 y. For stage IV, the filtering out of defects is appropriate (convex curve, sliding-τ, *b* > 0). The table shows a higher initial lifetime expectation for the new (2010–2014) compared to old (1989–1994) but a lower *b* and *R*-value (2.7 instead of 5.8) and surprisingly, the same long-term dynamic lifetime of 3.7 y. This points to an improved treatment and an improved statistical homogeneity in the new group compared to the old group, perhaps due to earlier diagnosis.

12. Study by Chang et al. [16] on advanced pancreatic cancer treated with gemicitabine plus S-1.

**Figure 11:**
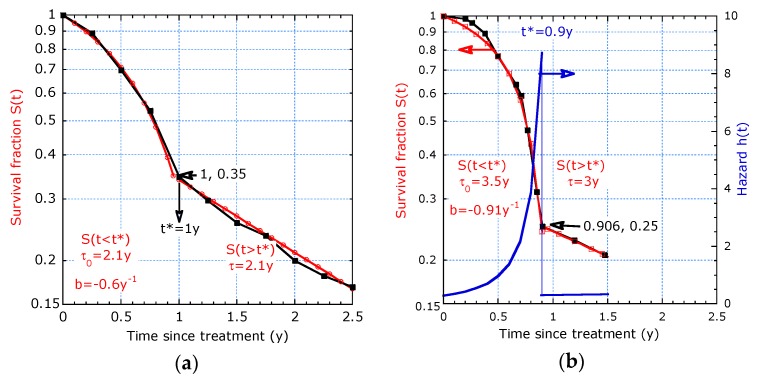
The survival curves (solid squares) from [16] with a clear transition time t* and curve fits (open squares) in distinct time intervals, starting with accelerated aging and switching abruptly to a lower constant hazard. The log-lin presentation reveals t* and the fit parameters before and beyond t*: (**a**) Figure 11a is from the complete group (111 patients) ([16], Figure 1); (**b**) Figure 11b is from 33 patients stratified by prognostic group (intermediate) [16]. The right scale shows the discontinuity in the hazard.

The overall survival plot of patients in Figure 11 shows a typical transition time, t*, where a sharp change in slope occurs. The results are neither to be fitted over the whole observation time with one of the proposed models nor with the piecewise linear model. The plot starts with a concave curve and ends with a straight line. The concave part is fitted with accelerated aging in the interval 0 < *t* < t*. The straight line is fitted with the exponential decay in the interval t* < *t* < *t_max_*. The sudden fall in risk to a much lower value for the patients surviving 1 year may be due to a typical biologic process in advanced pancreatic cancer with this treatment.

### 3.2. Merits of Providing Dynamic Lifetime

A few studies are highlighted to illustrate the added value of providing dynamic lifetime data together. Table 1 compares data from Kuijpers et al. [9], Glehen et al. [10] and Orsini et al. [4]. In [9,10], no selection with respect to tumour stage was made and the results obey the sliding-τ model. In [4], a relative survival study on patients with rectal cancer between 1989 and 2010 was made. The patients were selected on the pathological stage and their age. In that case, an exponential decay holds. The last column shows the ratio *R* between short- and long-term dynamic lifetime.

Survival data in tables after only 1, 3 and 5 years as provided in [4] are insufficient for a reliable logarithmic plot and curve fit with two fit parameters. Therefore, the data can better be completed with a more sensitive dynamic lifetime. The target time of *τ_dyn_* is denoted as an argument, and the adjacent times at which the data are taken are denoted as the index of *τ*. Table 2 shows the calculated values for *τ*_0,1_ (0.5y), *τ*_1,3_ (2y), and *τ*_3,5_ (4y) with (13) applied to *S* (0y), *S* (1y), *S* (3y) and *S* (5y) from Orsini et al. [4]. The dynamic lifetime improves with observation time. The increase of *τ*_0,1_(0.5) between 1989–1994 and 2001–2010 points to an improved treatment of patients. The slight decrease of the ratio *τ*_3,5_ (4)/*τ*_0,1_ (0.5) in more recent time periods compared to the older time period is comparable to the trend in Figure 10b between new and old for stage IV in [15].

In Table 3, the same methodology is applied on data from Nafteux et al. [17] on surgery of the adeno-carcinoma of the esophagus of 1639 patients. Only three data points: *S* (1), *S* (3), and *S* (5) are mentioned. Therefore, *τ*_0,1_ (0.5), *τ*_1,3_ (2) and *τ*_3,5_ (4) are calculated for Table 3. For almost all patient selections it holds that the *τ*_0,1_ (0.5) is larger than *τ*_1,3_ (2). This seems not related to “accelerated aging” at the very start, but may be a consequence of excluding all 90-day mortalities in the international study [17]. Such strong censoring can increase the *τ*_0,1_ (0.5) value significantly. For all cases in Table 3, the dynamic lifetime at 4 years exceeds the value at 2 years, which points again to “filtering out of defects”. As expected, the 2001–2013 group has higher dynamic lifetimes than the 1990–2000 group.

## 4. Discussion and Conclusions

A presentation of survival data as the logarithm of the survival versus time has substantially added value in analysing cancer data. It informs immediately on evolving lifetime expectancy. Applying parametric models helps in quantifying these dynamics in terms of short and long-term lifetime expectation and is a valuable addition to providing the mean value. A few options are proposed which can account for either increasing or decreasing lifetime expectancy, leading to convex or concave logarithmic survival plots, respectively. Besides the established Weibull and piecewise linear models, this paper proposes two novel approaches based on: two-τ and sliding-τ modelling. All these models aim to account for a dynamic lifetime expectancy when a simple exponential decay does not hold.

Deviations from linear behaviour often point to a statistical inhomogeneity in the group. This occurs e.g., by mixing up tumour stages or types of metastases or different treatments. The parameters in the proposed sliding *τ*-model and two-τ model are capable of detecting and quantifying these effects.

## Figures and Tables

**Table 1:** Studies in terms of *T*_1/2_, *τ_dyn_* (0) = *τ*_0_, *b* and *R* for: (i) HIPEC selected on PC and PMP from [9]; (ii) PC from [10]; and (iii) relative survival *S_c_* (*t*) = *S_co_*/*S_o_* of the rectal cancer from [4].

Reference and *t_max_*		*T*_1/2_ [y]	*τ_dyn_*(0) [y] ^1^	*b* [y^−1^]	*R* (Δ*R/R*) ^2^
[9] 9 y, PC:	OS	2.6	3.5 (0.3)	0.05 (0.02)	2.1 (25%)
	PFS	1.2	1.3 (0.1)	0.25 (0.05)	10.5 (27%)
[9] 10 y, PMP:	OS	>10	11.8 (0.9)	0.06 (0.01)	2.56 (12%)
	PFS	6	4.6 (0.3)	0.13 (0.02)	5.3 (17%)
[10] 5 y:	OS	2.9	3.26 (0.07)	0.101 (0.009	2.2 (6%)
[10] 5 y:	DFS	1.2	1.37 (0.05)	0.25 (0.02)	5 (9%)
[4] 5 y:	stage II	>5	15 (2)	0	1 (-)
[4] 5 y:	stage IV	1.2	1.6 (0.1)	0	1 (-)

^1^ Values between brackets are one standard deviation margin; ^2^ The relative uncertainty (expressed as percentage) is calculated from the uncertainty in *b* according to Δ*R/R* = 2*t_max_*Δ*b*/(1 + *bt_max_*).

**Table 2:** Comparison between young and middle-aged patients with rectal cancer in ([4], Table 3).

Diagnosis Period	1989–1994		1995–2000		2001–2010	
Age Group (y)	≤40	41–70	≤40	41–70	≤40	41–70
short-term *τ*_0,1_ (0.5y) (y)	5.3	5.7	6.6	6.6	7.8	8.6
*τ*_1,3_ (2y) (y)	6.8	7.35	6.8	8.52	9.32	10.8
long-term *τ*_3,5_ (4y) (y)	11.4	11.8	15.0	12.6	14.8	15.4

**Table 3:** Calculated *τ*_0,1_ (0.5), *τ*_1,3_ (2) and *τ*_3,5_ (4) in months from OS data by Nafteux et al. ([17], Table 1).

Clinical Data	Median	*τ*_0,1_ (0.5y)	*τ*_1,3_ (2y)	*τ*_3,5_ (4y)
female	75	109	83	123
male	59	75	66	140
age < 60 y	108	107	72	161
age 60–75 y	61	73	72	138
1990–2000 period	43	58	58	110
2001–2013 period	96	100	77	167
